# Interaction between Fungal Communities, Soil Properties, and the Survival of Invading *E. coli* O157:H7 in Soils

**DOI:** 10.3390/ijerph17103516

**Published:** 2020-05-18

**Authors:** Guannan Huang, Jiafen Liao, Ziming Han, Jiahang Li, Liyue Zhu, Guangze Lyu, Lu Lu, Yuang Xie, Jincai Ma

**Affiliations:** 1 Key Laboratory of Ground Water Resource and Environment, Ministry of Education, Jilin University, Changchun 130021, China; huanggn17@mails.jlu.edu.cn; 2 Jilin Provincial Key Laboratory of Water Resources and Environment, Jilin University, Changchun 130021, China; liaojf18@mails.jlu.edu.cn (J.L.); lyzhu17@mails.jlu.edu.cn (L.Z.); lvgz19@mails.jlu.edu.cn (G.L.); lul19@mails.jlu.edu.cn (L.L.); xieya19@mails.jlu.edu.cn (Y.X.); 3 State Key Laboratory of Environmental Aquatic Chemistry, Research Center for Eco-Environmental Sciences, Chinese Academy of Sciences, Beijing 100085, China; hanziming18@mails.ucas.ac.cn (Z.H.); Lijiahang96@163.com (J.L.)

**Keywords:** *E.coli* O157:H7, survival, fungal community, soil, structural equation model

## Abstract

Pathogens that invade into the soil cancontaminate food and water, andinfect animals and human beings. It is well documented that individual bacterial phyla are well correlated with the survival of *E. coli*O157 (EcO157), while the interaction betweenthe fungal communities and EcO157 survival remains largely unknown. In this study, soil samples from Tongliao, Siping, and Yanji in northeast China were collected and characterized. Total DNA was extracted for fungal and bacterial community characterization. EcO157 cells were spiked into the soils, and their survival behavior was investigated. Results showed that both fungal and bacterial communities were significantly correlated (*p* < 0.01) with the survival of EcO157 in soils, and the relative abundances of fungal groups (Dothideomycetes and Sordariomycetes) and some bacterial phyla (Acidobacteria, Firmicutes, gamma- and delta-Proteobacteria)weresignificantly correlated with *ttd*s (*p* < 0.01). Soil pH, EC (electric conductance) salinity, and water-soluble nitrate nitrogen were significantly correlated with survival time (time to reach the detection limit, *ttd*) (*p* < 0.05). The structural equation model indicated that fungal communities could directly influence *ttd*s, and soil properties could indirectly influence the *ttd*s through fungal communities. The first log reduction time (*δ*) was mainly correlated with soil properties, while the shape parameter (*p*) was largely correlated with fungal communities. Our data indicated that both fungal and bacterial communities were closely correlated (*p* < 0.05)with the survival of EcO157 in soils, and different fungal and bacterial groups might play different roles. Fungal communities and bacterial communities explained 5.87% and 17.32% of the overall variation of survival parameters, respectively. Soil properties explained about one-third of the overall variation of survival parameters. These findings expand our current understanding of the environmental behavior of human pathogens in soils.

## 1. Introduction

Concerns about *E. coli* O157:H7 (EcO157) began with an outbreak of food-borne illness in the United States in 1982 [1].In the following years, more cases of EcO157 infectionshave been reported worldwide [2]. It was suggested that undercooked beef was the main cause of EcO157 infection [3]. However, there is growing evidence that fresh fruits and vegetables are the primary cause of EcO157 infection outbreaks [4,5]. The organic manure applied into the organic farms might contain a large number of EcO157,which could be transferred to surface run-off, leached intothe soil, and associated with plant roots [6]. EcO157 cellscould be attached to and even incorporated into fresh produce or plant tissues [7]. Previous studies have revealed that soilsmight be a major reservoir of EcO157, and this pathogen could survive there for weeks, even months. The extendedpersistence of EcO157 could significantly increase its chance to enter the food chain, and finally constitutelarge outbreaks of infection [8]. Therefore, understanding theirpersistencecould provide useful information in order to reduce the potential health risks associated with this pathogen.

Both abiotic and biotic factors could affect the persistence of EcO157 in soils in addition to its own biochemistry and genetic background [9,10,11,12]. Soil abiotic factors, including clay minerals, organic matters, temperature, pH, oxygen, and salinity, play a major role in controlling the survival of EcO157 in soils [13,14,15,16,17,18,19,20]. On the other hand, soil biotic factors, including soil microbial community, enzyme activities, and biofilms, are also critical for the fate of EcO157 in the environment [21,22,23,24,25]. 

Soil microbes, such as bacteria [26], virus [27], protozoa [28], are closely related to the survival of EcO157 [28,29,30,31]. Our previous study indicated that some specific groups of bacteria well linked with the persistence of EcO157 in soils, e.g., the survival time of EcO157 was positively correlated with the abundances of Actinobacteria (*p* < 0.001) and Acidobacteria (*p* < 0.05), while negatively correlated with the relative abundances of Proteobacteria and Bacteroidetes (*p* < 0.05) [26]. However, fungal communities, as a major part of the soil microbial flora, couldaffect the survival of EcO157 in soilsdue to their unique ecological role in soil ecosystems.Some fungi could produce extracellular enzymes thatcould degrade organic compounds, even highly recalcitrant carbonin the environment [32]. Therefore, fungi is a decomposer in the biosphere, and it contributes extensively to a wide range of biogeochemical cycling of nutrients, e.g., organiccarbon, nitrogen and phosphorus, which could be better nutrition for heterotrophs [33]. Reports of bacteria on the survival of EcO157 are well documented. However, it is still unclear that how fungal communities affect the survival of the invading human pathogens, e.g., EcO157. Thus, the role of soil indigenous fungal communities on the survival of EcO157 deserves additional investigation, especially in the presence of other microbial communities.In the current study, soils of different land-use types were selected, fungal and bacterial community composition and structure werecharacterized, EcO157 was spiked into those soils;in this way, the influence of fungal communities on the survival of EcO157 was investigated [34]. Special focus was put on the role of fungal composition and structure on the persistence of EcO157 in soils when the indigenous bacterial communities were taken into account. 

Tongliao (TL), Siping (SP), and Yanji (YJ) are typical areas of 3 major land-use types in northeasternChina.TLsoilswerefroma typical agricultural–pastoral land in the west of Jilin Province. In such land-use types, cow manure and beef products might be the source of EcO157 [35].Land use types in Siping and Yanji are vegetable land and artificial economic forestland (apple–pear growing farm), respectively. Potential EcO157 source of SP and YJsoilsmay include the application of poorly composed organic fertilizer, feces of local livestock farms and wild animals, run-off from the piles of animal wastes, and pathogen-containing dust deposited into the land. Fresh vegetables and fruitsare important directions for the prevention and control of food-borne pollution [36].Microbial community structure and composition, as well as the soil physicochemical properties, might vary significantly in soils from different land-use types [37,38], and such variation might, in turn, influence the persistence ofpathogens invaded into those soils.However, the influence of soil abiotic and biotic factors on the survival of the invading human pathogensremains poorly understood.

In this study, an in-lab experiment was conducted to simulate human pathogen invasion into the soils by spiking EcO157 cells into soils from Tongliao (TL), Siping (SP), and Yanji (YJ). An array of statistical analyseswere performed to probe the potential interaction between the start point fungal communities, bacterial communities, soil properties, and the survival parameters. The objectives of the study are to (1) investigate the survival behavior of EcO157 in soils, and (2) analyze the linkage between EcO157 survival parameters and fungal/bacterial communitiesand soil parameters.

## 2. Materials and Methods

### 2.1. Soil Sample Collection and Characterization

Twenty-one samples were collected in northeastern China (8 from Tongliao, 6 from Siping, and 7 from Yanji). Each sample (0–20 cm) was a composite of 3 individual soil cores taken at 5-m intervals, and triplicate samples were taken. Fresh soil samples were packed in plastic bags and transported to the labon ice. Soils were sieved (<2 mm)and stored under4 °Cuntil use after the removal of plant debris, roots, and stones.A small portion of sampleswas saved under −80 °C for DNA extraction. Subsoil samples were air-dried under room temperature (20 ± 1 °C)forsoil physicochemical properties determination. Soil propertiesare shown in Table S1.

### 2.2. Soil Inoculation and Enumeration

The stock EcO157 EDL931 (ATCC 35150) from−80 °C freezer was inoculated onto a fresh LB (Luria-Bertani) agar plate and incubated at 37 °C for 14–16 h [39]. A single colonywas picked up and inoculated on a solid medium containing two antibiotics (nalidixic acid 20 mg/L and rifampicin 100 mg/L) at 37°C for 14–16 h. A single colony was selected to grow to a larger volume in LB medium; then, the cells were washed twice using 0.9% NaCl solution and resuspended in deionized water to reach OD_600nm_ = 1.0 for inoculation into the soilsamples.EcO157 cells were added into soils to a final density of about 0.5 × 10^7^ CFU per gram soil (gdw^−1^), and the moisture content of all the soil was adjusted to 60% of water holding capacity (WHC) using sterile deionized water. Briefly, the cell suspension was thoroughly mixed with soil in a plastic bag, and 500 g of the inoculated soil was transferred to a top perforated plastic bag for air exchange [22]. The same amount of non-inoculated soil was put into another plastic bag, which was used as uninoculated control with deionized water being added instead of cell suspension. The control experiments were used for the determination of moisture loss and a check of potential contamination. The moisture content was adjusted to 60% of water holding capacity (WHC) across all soil samples by adding deionized water. All the spiked soil samples were prepared in triplicateand saved in the dark at room temperature (20 ± 1 °C). Duplicate soil samples (about 1.0–1.2 g in test tubes) were taken periodically from each of the bags using a sterile spoon. The EcO157 cells were extracted with 0.1% buffered peptone water (Lab M, Lancaster, UK) to determine the survivors. In brief, a 4.0 mL of 0.1% peptone buffer (Lab M, Lancashire, UK) was added to the test tube containing the soil sample, and the soil was thoroughly mixed with the buffer by inverting the tube several times and then vortexed for 2 × 20 s. The resulting soil paste (cell suspension) was then subjected to 10-fold serial dilutions. Fifty μL of the two highest dilutions were plated in duplicate on SMAC/BCIG agar (Lab M, Lancashire, UK) with appropriate antibiotics (nalidixic acid 20 mg/L and rifampicin 100 mg/L) for enumeration. The inoculated SMAC agar plates were incubated at 37 °C for 16 h to allow colony development.Colonies formed on the SMAC agar werecounted, and the results were expressed as colony-forming units per gram dry weight (CFU ·gdw^−1^). Our preliminary experiments showed that the average cell recovery rate of the method was >90% [40]. Samples were continually taken until the cell concentrations drop belowthe detection limit (100 CFU·gdw^−1^).

### 2.3. Soil DNA Extraction, Illumina MiSeq and Sequence Data Analysis

DNA extraction quality was determined using 0.8% agarose. Total DNA was extracted from 0.5 g of soil using a DNA isolation kit (Omega, Norcross, GA, USA), quantified using NanoDrop NC 2000 (Thermo Scientific, Waltham, MA USA), and the quality of DNA was assayed on a 0.8% agarose gel via electrophoresis. For fungal and bacterial community characterization, the fungal ITS gene was amplified by using the primer set, including the forward primer 1737F (5’-GGAAGTAAAAGTCGTAACAAGG-3’) and the reverse primer 2043R (5’-GCTGCGTTCTTCATCGATGC-3’); for the bacterial community, the 16S rDNA amplicon library construction, primers 338 (50-ACTCCTACGGGAGGCAGCA) and 806R (50-GGACTACH VGGGTWTCTAAT) were used for amplifying hypervariable regions V3 and V4. The purified samples were sent to Shanghai Personal Biotechnology Co., Ltd. for sequencing using the Illumina MiSeq high-throughput sequencing platform. The QIIME software (Quantitative Insights Into Microbial Ecology, v1.8.0, http://qiime.org/) was used to identify the question sequence [38]. Subsequently, USEARCH (v5.2.236, http://www.drive5.com/usearch/) was called through the QIIME software (v1.8.0, http://qiime.org/) to check and eliminate the chimeric sequences. The results obtained by sequencing were sequence filtered using QIIME. OTU (operational taxonomic unit) cluster analysis used the Uclust method in QIIME to cluster high-quality sequences by 97% similarity and selected the longest sequence of each class as the representative sequence. The taxonomic information of each OTU was obtained by comparing the sequence databases using the BLAST method in QIIME.

### 2.4. Survival Data Modeling

The survival of EcO157 was analyzed by fitting the survival data to the Weibull model using GinaFitv1.5 [41]. The Weibull model was constructed based on the hypothesis that the deactivation kinetics of the EcO157 population follows a Weibull distribution [42]. Survival parameterscould be calculated using the following equation:

(1) $$\log\left(N_{t}\right)=\log\left(N_{0}\right)-{\left(t/\delta \right)}^{p}$$

where *N* is the number of survivors, *N*_0_ is inoculums size, *t* is time (days) post-inoculation, and *p* is the shape parameter; when *p* > 1, it is a convex curve, when *p* < 1, it is a concave curve, and when *p* = 1, it is linear. The scale parameter *δ* represents the time required for the bacterial concentration to decrease by an order of magnitude. Time needed to reach the detection limit (*ttd*) can also be calculated when using GInaFiT to fit the experimental survival data. 

### 2.5. Statistical Analysis. 

Graphics were conducted with Origin 9.0 (Origin Lab Corp, Northampton, MA, USA). Dissimilarity analysis, the Mantel test, multiple regression on distance matrices (MRM), and principal coordinate analysis (PCoA) were performed using R 3.5.2 (http://www.R-project.org) with vegan and ecodistpackages. One-way ANOVA for survival parameter comparison and Pearson correlation between soil properties and fungal classes was performed with SPSS 22.0 (IBM, Armonk, NY, USA).The Mantel test and partialMantel test, MRM, and variation partition analysis (VPA) in the vegan package were used to delineate the effects of soil properties and fungal/bacterial community on survival parameters (*ttd*, *p*, and *δ*). The structural equation model (SEM) was performed with AMOS 22.0 software (Amos Development, Spring House, PA, USA) to evaluate the direct and indirect effect of fungal communities and soil properties on survival parameters.

## 3. Results

### 3.1. Fungal Community Composition and Structure

**Figure 1:**
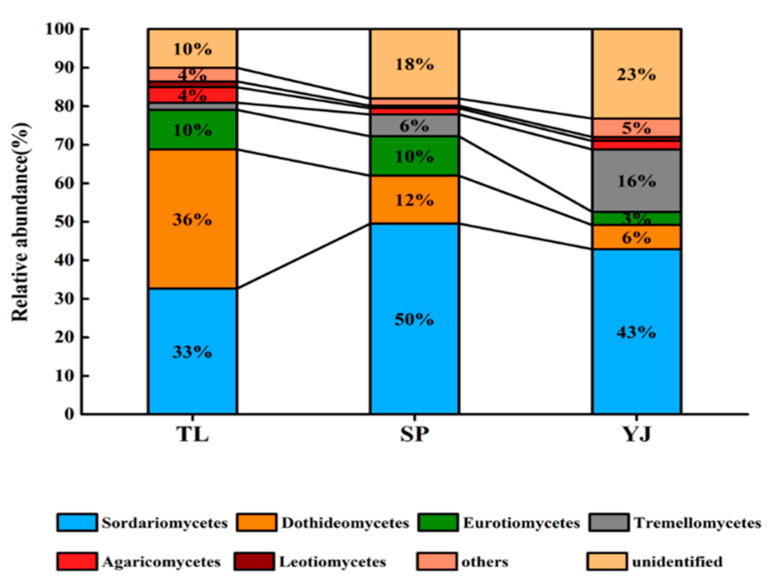
The columnar stacking diagram showing the relative abundance (%) at the class level of fungal communities. TL, SP, and YJ indicate that the soil samples were collected from Tongliao, Siping, and Yanji, respectively.

The composition and relative abundances of fungal communities in soilsfrom TL, SP, and YJ are shown in Figure 1. Subclasses (relative abundance >1%) of phylum Ascomycota were plotted, i.e.,Sordariomycetes, Dothideomycetes, Eurotiomycetes, Tremellomycetes, Agaricomycetes, Leotiomycetes, of phylum Ascomycota.The most abundant fungal group in TL soilswas Dothideomycetes, while in SP and YJ soils, the abundance of Sordariomycetes was the highest. Overall, fungal composition and structure in SP and YJ soils shared more similarities, in contrast to that in TL soils.

**Figure 2:**
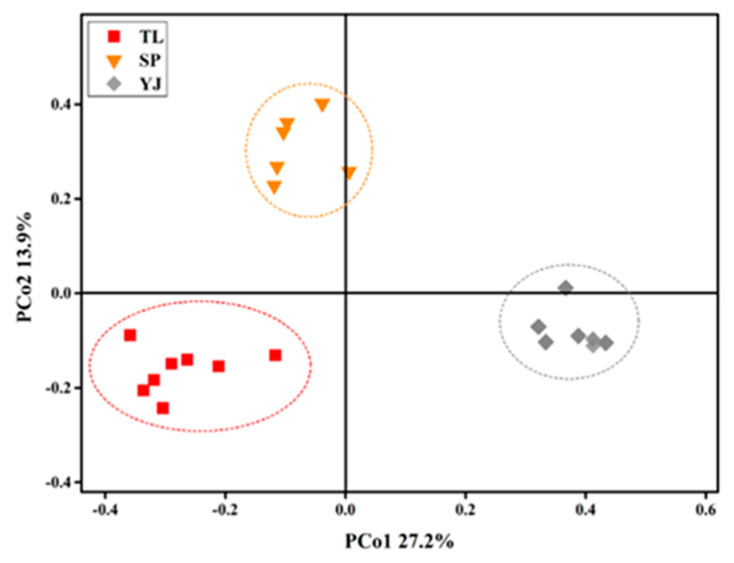
Result of principal coordinate analysis (PCoA) of fungal communities in soils from different sampling sites. TL, SP, and YJ indicate that the soil samples were collected from Tongliao, Siping, and Yanji, respectively.

PCoA (principal coordinate analysis) was performed based onthe Bray–Curtis dissimilar matrix of the soil samples (Figure 2). Soil samples from the same sampling site wereclustered together, indicating that the fungal community structure in soilsfrom the same sampling sitesshared more similarities. Further dissimilarity analysis based on different distance indicators revealed that fungal community structure in soils from different sampling sites varied significantly (*p* < 0.05; Table 1). PCo1 and PCo2 co-explained 41.1% of the overall variation of the fungal community in all soil samples.

### 3.2. Comparison of Survival Profiles and Survival Parameters (ttd, p, and δ).

**Figure 3:**
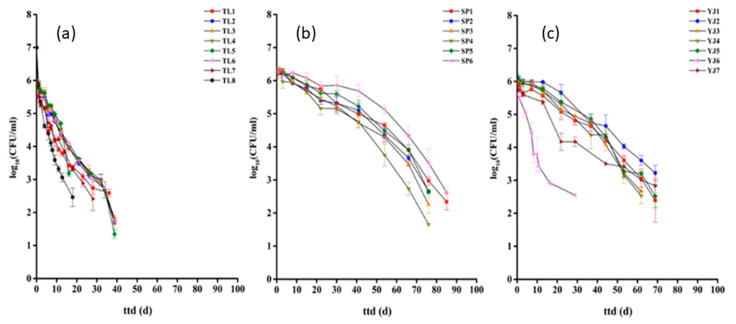
Survival profiles of *E. coli* O157:H7 in soils from different sampling sites. TL (**a**), SP (**b**), and YJ (**c**) indicate that the soil samples were collected from Tongliao, Siping, and Yanji, respectively. Standard errors were means of triplicate measurements.

**Figure 4:**
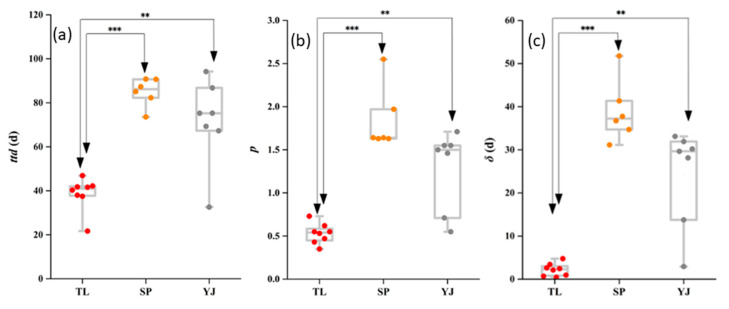
Comparison of survival parameters *ttd* (**a**), *p* (**b**), and *δ* (**c**) of *E. coli* O157:H7 in soils from different sampling sites. TL, SP, and YJ indicate that the soil samples were collected from Tongliao, Siping, and Yanji, respectively. *, **, and *** indicate statistical significance at 0.05, 0.01, and 0.001 levels.

In general, survival profiles of EcO157 in TL soils displayed as concave curves, whilemore convex curves were observed in SP and YJ soils (Figure 3), and the overall number of EcO157 survivors tended to decline faster in TL soils than in SP and YJ soils (Figure 3). Further statistical analysis revealed that the average *ttd*s of EcO157 in TL, SP, and YJsoils were 38.75 days, 84.97 days, and 71.54 days, respectively. The *ttd*s of EcO157 in TL soil were significantly greater (*p* < 0.01) than those in SP and TL soils (*p* > 0.05). Interestingly, other survival parameters (*p* and *δ*) followed the same trend of *ttd* (Figure 4). It was found that a larger *ttd* of EcO157 was generally associated with a larger *δ*. For the shape parameter *p*, SPsoilsgenerally yielded a *p* greater than 1, while most of the *p*s in TL soilswere less than 1 (Figure 4).

### 3.3. Pearson and Mantel Correlation between Soil Properties, Survival Parameters, and Relative Abundances of Selected Fungal Classes

**Figure 5:**
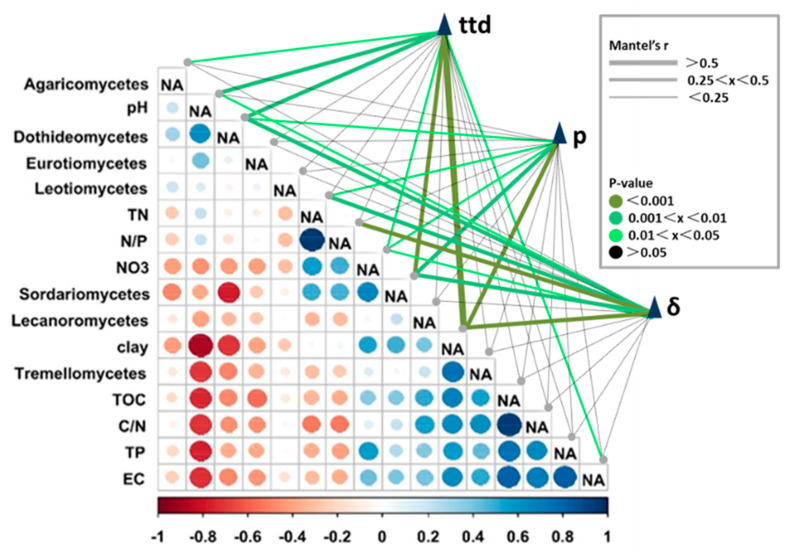
The bottom left diagram showing the Pearson correlation of environmental physicochemical parameters and relative abundances of fungal groups at class levels. A color gradient denotes the Pearson’s correlation coefficients. The upper right graph shows the Mantel test between survival parameters (*ttd*, *p*, and *δ*) and the soil properties mentioned above. EC, electrical conductivity; TOC, water-soluble organic carbon; TN, total soluble nitrogen; NH_4_-N, ammonium nitrogen; NO_3_-N, nitrate nitrogen; TP, total dissolved phosphorus; clay(%), soil clay content.

**Figure 6:**
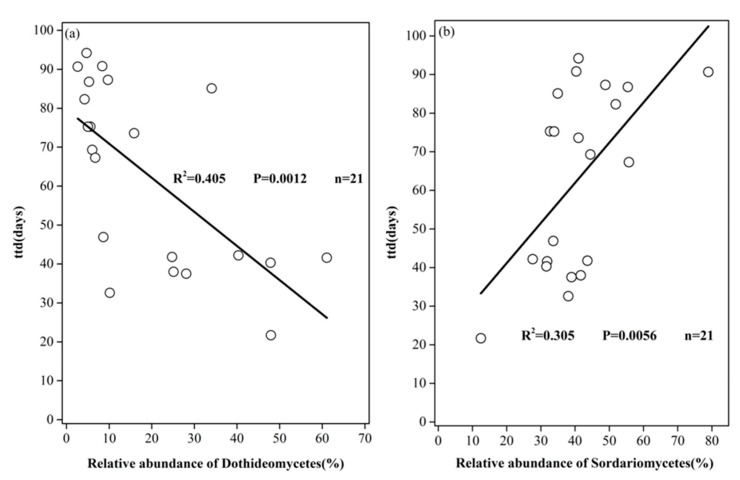
Linear regression of the relative abundances of Dothideomycetes (**a**) Sordariomycetes (**b**) and *E. coli* O157:H7 survival time (*ttd*s).

In order to explore the correlation between fungal communities and soil properties, the correlation between the relative abundance of the fungalgroups at the class level and the soil properties was established using Pearson correlation analysis (Figure 5 bottom left). A correlation was also established between the environmental factors and survival parameter matrices using the Mantel test (Figure 5, upper right). Pearson correlationshowed that pH was positively correlated with the relative abundances of Dothideomycetes and Eurotiomycetes, while negatively correlated with the relativeabundances of Sordariomycetes and Tremellomycetes. Clay content was negatively correlated with Agaricomycetes, Eurotiomycetes, Sordariomycetes, and positively correlated with Sordariomycetes and Tremellomycetes. Results of the Mantel test indicated that Dothideomycetes and Sordariomyceteswere significantly correlated with all the survival parameters. Further linear regression analysis confirmed thatthe abundance of Dothideomycetes class was significantly negatively correlated with *ttd* (*R^2^* = 0.4132, *p* < 0.01), while the abundance of Sordariomycetes class was significantly positively correlated with *ttd* (*R^2^* = 0.3888, *p* < 0.01) (Figure 6).

### 3.4. Correlation Analysis between Fungal Community, Bacterial Community, and Survival Parameters

Correlation analysis was performed, aiming to confirm whether there was a close relationship between fungal community, bacterial community, and EcO157 survival parameters (*ttd*, *p*, and *δ*). The results (Table 2) showed that the survival parameters matrix was well correlated with fungal and bacterialcommunities using both the Mantel test and multiple regressions on distance matrices (*p* < 0.05).

The Mantel test and a partial Mantel test between each of survival parameters (*ttd*, *p*, and *δ*) and dominant fungi and bacteria phylaorselected bacterial classes (gamma- and delta-Proteobacteria) were performed, aiming to determine which bacterial and fungal groups were significantlycorrelated with the survival parameters. A partial Mantel test could avoid the potential interference brought by other co-existed factors. The results (Table 3) showed that survival parameters (*ttd*, *p*, and *δ*) were well correlated with some fungal and bacterialgroups (*p* < 0.05). In partial Mantel test results, Dothideomycetes and Sordariomycetes were still significantly related to *ttd* and *p*, respectively, and both are significantly related to *δ*. Some bacterial communities were also significantly related to survival behavior, including Acidobacteria, Chloroflexi, gamma-Proteobacteria, and delta-Proteobacteria.

### 3.5. Structural Equation Model(SEM) and Variation Partition Analysis (VPA)

**Figure 7:**
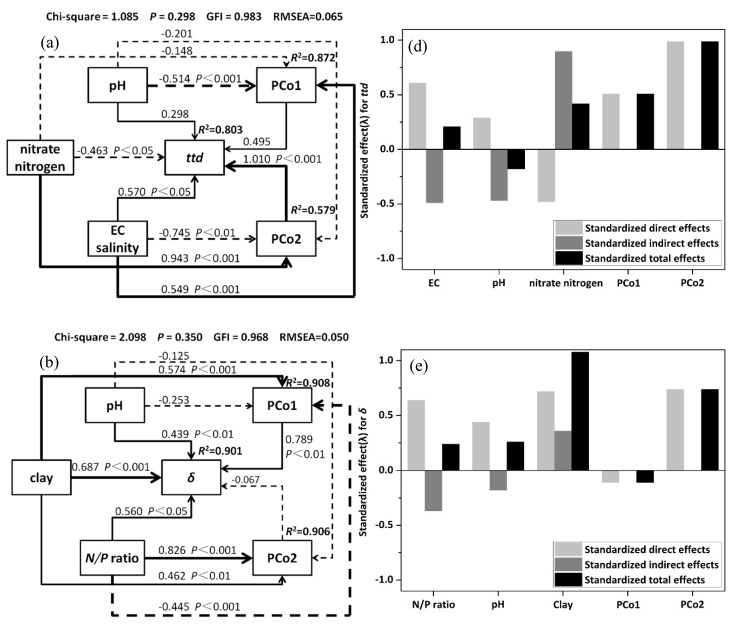

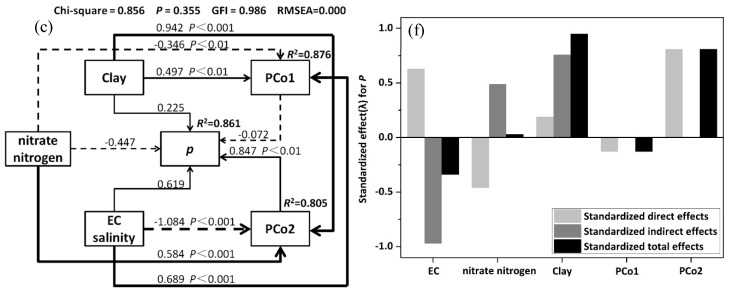
Structural Equation modeling (SEM) quantified the indirect and direct effects of soil properties and fungal communities on the survival parameters *ttd* (**a**,**d**), *δ* (**b**,**e**), and *p* (**c**,**f**) of *E. coli* O157:H7. The width of the arrow indicates the strength of the normalized path coefficient (λ). The solid and broken lines indicate the positive and negative path coefficient, respectively. The *R*^2^ value represents the proportion of the variance explained for each variable. The total effect was the sum of direct and indirect effects. The first 2 coordinates (PCo1, PCo2) of the principal coordinate analysis were used to represent the fungal community composition.

In order to predict the direct and indirect effects of soil fungal communities (represented by the first 2 axes of PCoA) and selected soil properties on the survival behavior of EcO157 (as indicated by *ttd*, *p* and *δ*), structural equation model was constructed.The results (Figure 7a) showed that the *ttd*s had direct links to the fungal community (PCo1), pH, and EC salinity. In addition, pH, nitrate nitrogen, and EC salinity indirectly correlated with *ttd*s throughthe fungal community (PCo1 and PCo2), and the overall indirect effect caused by soil properties was larger thanthe direct effect (Figure 7d).The*δ*swere directly correlated withpH, clay, and N/P ratio.Clay and N/P ratioalso indirectly correlated with *δ*s through the fungal community (Figure 7b). The overall direct effect outweighed the indirect effect (Figure 7e). It was also found that *p*s were directly linked to the fungal community, andno significant direct effect was found between *p*s and soil properties (clay, nitrate nitrogen, and EC salinity) that exerted more indirect effect on*ttd*s through the fungal community.

**Figure 8:**
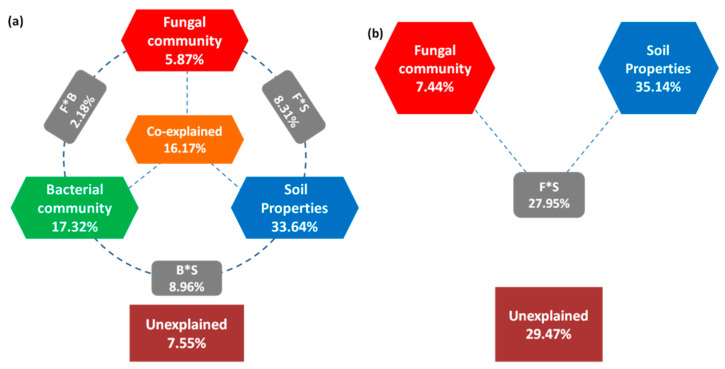
Variation partitioning analysis (VPA) of survival behavior of *E. coli* O157:H7 explained (%) by soil property, fungal community, and bacterial community (**a**) and soil property, fungal community (**b**).

Variation partition analysis (VPA) was performed to probe to what extend fungal communities influenced the survival behavior of EcO157. The survival parameters (*ttd*, *p*, and *δ*) generated from each soil were used to construct the independent matrix, the soil properties and the OTUs’ matrix of the fungal and bacterial communities were used to construct the dependent matrices. The contribution of the fungal community, bacterial community, and soil properties to the overall variation in EcO157 survival behavior (*ttd*, *p*, and *δ*) were calculated. Soil properties were the most important influencing factors, followed by soil bacterial (Figure 8a) and fungal communities (Figure 8b), in shaping the survival of EcO157 in soils. Soil properties explained 35.41% of the overall variation of the survival parameters of EcO157. Fungal communities explained 7.44% of the overall variation of survival parameters without consideration of bacterial communities, and this number slightly dropped to 5.87% when bacteria were taken into account. In contrast, bacterial communities explained 17.32% of the overall variation of the survival of EcO157 in soils. 

## 4. Discussion

Our results (Figure 1 and Figure 2, Table 1) collectively showed that the fungal community structure and composition varied significantly among the soils collected from TL, SP, and YJ. Such variation might be due to the difference in local climateor land-use types [43,44], geographical distances, and the localclimatic conditions, e.g., mean annual temperature and mean annual precipitation [45]. It has been reported that major fungal phyla in global soils were Ascomycota and Basidiomycota, and the relative abundances of Ascomycota and Basidiomycota displayed major differences with respect to land types [46].

Results (Figure 3 and Figure 4) showed that EcO157 displayed different survival profiles in TL soils compared with those in SP and YJ soils. Overall, EcO157 showed more persistence when inoculated in SP and YJ soils than in TL soils. It was speculated that such variation might result in the differences in soil physicochemical parameters and biotic factors. Our previous data and other’s studies revealed that soil physical properties could exert significant influence on the survival of EcO157 in soils, e.g., clay content and EC salinity; soil chemical properties including pH, organic carbon, and nitrogen; soil biotic factors, e.g., bacterial community [22,24,26]. It was proposed the survival of EcO157 in soils was associated with the land use types; the *ttd*s were relatively short in infertile soil [47], which is consistent with our current study as TL soils were relatively barren compared with SP and YJ soils.

Our results showed that pH, nitrate nitrogen, water-soluble organic carbon, and clay content might be major factors shaping the fungal community (Figure 5), which was consistent with previous results showing that pH and organic carbon were the major factors shaping the overall fungal community in soils [43,48]. It was well established that the organic matter amendment could promote fungal abundance by optimizing soil structure [49]. In addition, soil salinity, land use type might also have a major influence in shaping soil fungal community composition and structure [50,51]. Recent studies revealed that soil pH was also an important environmental filter for the fungal community [52,53]. Land-use type or land management history also plays some role in fungal community structure [38,43].

Results of linear regression analysis and the Mantel test showed that the relative abundances of Dothideomycetes class and Sordariomycetes class werewell correlated with *ttd*s (*p* < 0.01), respectively (Figure 5 and Figure 6, and Table 3). Dothideomycetes [54,55] and Sordariomycetes [56] are the dominant microorganisms of Ascomycota. Some cultivable fungi have been isolated, and most of them were Ascomycota, showing antibacterial activity against *E. coli* [57]. Dothideomycetes might be the fungus that negatively influencesthesurvival of *E. coli*; however, Sordariomycetes might play a major role in improving the survival of EcO157 in soils by degrading high-molecular-weight organic compounds into readily available organic carbon and nitrogen nutrients for EcO157. Generally, fungi are the main decomposers of the remains of animals and microorganisms [58], and there is a strong correlation between saprophytes and soil nutrient levels [59]. Previous studies have shown that some fungi can produce toxins and antibiotics that mayplay a negative role in the survival of EcO157 [60].

The Mantel test (Table 3) also illustrated that bacterial groups, e.g., Acidobacteria and gamma-Proteobacteria, were well linked to the survival parameters, which was well in line with our previous studies [26]. Our previous results showed that Acidobacteria and gamma-Proteobacteria were positively and negatively correlated with the survival of EcO157, respectively [26]. Acidobacteria may improve the survival of EcO157 by degrading higher molecular weight organic compounds into smaller organic carbon molecules thatcan be utilized by EcO157 cells as carbon sources for growth and survival [61]. Whole-genome analysis revealed that Acidobacteria is capable of breaking down plant polymers [62]. On the other hand, the correlation betweengamma-Proteobacteria and EcO157 may be explained by the strong competition for nutrients and habitats within the same niche. As a member of gamma-Proteobacteria, EcO157 shares many biochemical, physiological, and ecological similarities with other members fromgamma-Proteobacteria.A report showed that the survival of EcO157 could be suppressed by gamma-Proteobacteria in livestock bedding [63].

Our results showed that the soil fungal communitymatrix (including all fungal OTUs)was significantly correlated with the survival behavior matrix (*ttd*, *p*, and *δ*) of EcO157 in soil (Table 2), and different fungal groups (at class levels) exerted different influences on the survival parameters of EcO157 (Figure 6), indicating that overall fungal community has a direct effect on the survival of EcO157 in soils, and distinct mechanisms might be involved in the persistence of this pathogen in soils. Among the survival parameters, survival time (*ttd*) was the most important factor in evaluating the health risks associated with EcO157. Results of the SEM analysis (Figure 7) revealed that in addition to the direct impact by the fungal community on *ttd*s, soil properties (EC salinity and nitrate nitrogen) could indirectly influence the *ttd*s through the fungal community. Effect of soil physical (e.g., clay content and EC salinity) and chemical properties (e.g., pH, assimilable organic carbon) on the survival of EcO157 had been extensively reported [14,22,24,26,64], while the indirect effect of soil properties on the survival of EcO157 through the fungal community was firstly reported by the current study. The direct effect of the fungal community might be explained by the interaction between fungi and EcO157 as discussed before, while the indirect linkage between soil parameters (e.g., EC salinity, pH, and nitrate nitrogen) via fungal community could be explained by the close correlation between soil properties and fungal community. It was reported that salinity might change fungal community structure, and result in the increase of the abundance of Ascomycota [65], and fungal diversity was represented mostly by Dothideomycetes members in high salinity conditions [66], EC salinity might contribute to the variation in trophic guilds [67]. Previous studies showed that when nitrate-nitrogen concentration was high, Sordariomycetes and Eurotiomycetes dominated the fungal community [68]; similarly, the abundance of Sordariomycetes was also linked to nitrate nitrogen levels. Reports showed that some fungi provided denitrification effect in addition to nitrate mineralization and heterotrophic denitrification [69,70], and members of a subclass of Sordariomycetes displayed the N_2_O-producing activities [71]. The denitrification of fungi might relieve the toxic effect caused by high levels of nitrate in soils [72], thus might promote the survival of bacterial pathogens in soils. The pH was also a major regulator in shaping the fungal community structure and composition [73], and soil fungal community composition was largely controlled by pH. It seemed that the structure of soil fungal communities was the result of complex interactions among factors that might favor beneficial or detrimental relationships. It was true that the soil parameters could change the fungal community, and such variation could, in turn, influence the survival of EcO157 inoculated into the soils.

It was interesting that the first log reduction time, *δ*, was mainly influenced by soil physicochemical properties (pH, clay content, and N/P ratio), although fungal community also directly linked to *δ*. After EcO157 cellswere inoculated into the soil samples, they might struggle to survivein such a harsh environment. Compared to soil bacteria and fungi, soil properties might exert a more direct impact on the survival of EcO157 in soils, and such a conclusion could be supported by the closer links between *δ* and soil physical and chemical parameters (Figure 5). In contrast, the shape parameter, *p*, was only significantly linked to the fungal community, highlighting the important role of the fungal community on the determination of *p* values. Compared with *δ*, a parameter showing the goodness of initial survival phase of EcO157 in soils, *p* was more likely to represent the overall survival of EcO157 in soils, as it determined the shape of survival curves.

According to the data obtained in this study, the potential mechanisms that fungus influence the survival of EcO157 in soils might include (1) the direct effect of fungal community on the survival of EcO157 (this effect could well explain the variation in the shape (*p*) of the survival curves;the direct influence ofthe fungal community (structure and composition) could be positive, negative, or neutral effect brought by different fungal communities), and (2) the indirect influence of soil properties on the survival of EcO157 via the fungal community. Soil physical and chemical properties might change the composition and structure of the fungal community, and such change may, in turn, influence the survival of EcO157 in soils. In addition, the interactions between fungal and bacterial communities might also play an important role in controlling the survival of EcO157 in soils.

It should be noted that the survival behavior of EcO157 and other invading pathogens might be a function of both soil abiotic and biotic properties. The measured soil properties or combined soil properties could not fully explain the variation of survival behavior, which might be due to the unmeasured soil abiotic parameters, trace elements, anion exchange capacity, humus, soil minerals, as well assoil biotic factors, including soil bacteria, protists, viruses, biofilms, and soil animals. However, the inclusion of thecontrol experimentsusing sterilized soils is required in order to assess the fungal involvement and to distinguish direct and indirect effects brought by fungal communities. The survival mechanisms could not be clearly elucidated until all those data were available. It also should point out that the correlation analysis was only established between the start point fungal community and soil properties and the survival parameters. The composition and structure of the fungal community might vary during the course of the experiment, and the fungal community might evolve toward a similar direction under the same experimental conditions (temperature and moisture content), and such change could be linked to the survival of EcO157 in soils.

## 5. Conclusions

In summary, our results show that overall fungal communitiesare significantly correlated with the overall survival of EcO157 in soils, although they only explain a relatively small fraction of the overall variation of survival parameters of EcO157 in soils compared with bacterial communities. Twogroups of fungal communities differently influenced the survival of EcO157 in soils. Soil properties were the major determinants for the decay of *E. coli* O157 population in soils. Our study highlights the complex interaction between the survival of EcO157and the soil fungal and bacterial communities, as well as soil properties. Our results provide additional insights into the environmental behavior of EcO157 in a soil environment.

## Figures and Tables

**Table 1:** Dissimilarity analysis of fungal community structures in different types of soil. The distance metrics used were Bray, Jaccard, Horn, and Euclidean. Three distance indices were calculated, including mrpp, Adonis, and Anosim. Bolded numbers indicated *p* values were significant at the 0.05 level. MRPP, Multi Response Permutation Procedure; Anosim, Analysis of similarities.

Distance Method		MRPP		Adonis		Anosim	
		*δ*	*p*	*F*	*p*	*R*	*p*
site	Bray	0.6373	**0.001**	6.5737	**0.001**	0.8816	**0.001**
	Jaccard	0.7697	**0.001**	4.2375	**0.001**	0.8816	**0.001**
	Horn	0.5839	**0.001**	8.0129	**0.001**	0.7586	**0.001**
	Euclidean	8357	**0.001**	4.1364	**0.001**	0.3711	**0.001**

**Table 2:** Correlation analysis of fungal and bacterial community (OUT matrix) and *E. coli* O157 survival parameters (*ttd*, *p*, and *δ*) based on the Mantel test based on Pearson’s product–moment correlation and Spearman’s rank correlation rho and multiple regression on distance matrices (number of permutation=9999). Bolded numbers indicated *p* values were significant at 0.05 level.

Primary Matrix	Secondary Matrix	Correlation Methods						
		Mantel Test				Multiple Regression on Distance Matrices (MRM)		
		Pearson’s Product-Moment		Spearman’s Rank Correlation Rho				
fungal community	survival parameters	*r*	*p*	*r*	*p*	*R^2^*	*F*	*p*
		0.387	**0.002**	0.3857	**0.003**	0.1498	36.6372	**0.002**
bacterial community		*r*	*p*	*r*	*p*	*R^2^*	*F*	*p*
		0.5174	**0.001**	0.4399	**0.001**	0.2677	72.0259	**0.001**

**Table 3:** Mantel test and partial Mantel test between survival parameters (*ttd*, *p*, and *δ*) and dominant fungal and bacterial phyla and classes. Fungal groups were shown in bold, numbers in bold indicate correlation was significant at 0.05 level.

Survival Parameter	Bacterial/FungalGroups	Controlling for	Mantel		Partial Mantel	
			*r*	*p*	*r*	*p*
*ttd*	Acidobacteria	other bacteria and fungi	0.1922	**0.024**	0.0836	0.097
	Actinobacteria		0.1694	0.055	0.0171	0.552
	Chloroflexi		0.1963	**0.015**	0.2010	**0.026**
	Firmicutes		0.2886	**0.003**	0.1976	**0.024**
	Gemmatimonadetes		0.0430	0.265	0.0120	0.364
	Delta-Proteobacteria		0.3316	**0.004**	0.3272	**0.006**
	Gamma-Proteobacteria		0.3027	**0.002**	0.1844	**0.024**
	**Dothideomycetes**		0.3435	**0.003**	0.2991	**0.003**
	**Sordariomycetes**		0.2104	**0.022**	0.1308	0.076
*p*	Acidobacteria		0.2499	**0.016**	0.1668	**0.033**
	Actinobacteria		0.1793	0.055	0.0234	0.331
	Chloroflexi		0.1351	0.058	0.1358	0.083
	Firmicutes		0.1495	0.096	0.0523	0.224
	Gemmatimonadetes		0.3775	**0.017**	0.3672	**0.028**
	Delta-Proteobacteria		0.2164	**0.011**	0.2064	**0.018**
	Gamma-Proteobacteria		0.3912	**0.001**	0.3123	**0.002**
	**Dothideomycetes**		0.1922	**0.048**	0.1410	0.072
	**Sordariomycetes**		0.4177	**0.004**	0.1922	**0.050**
*δ*	Acidobacteria		0.2358	**0.008**	0.1512	**0.039**
	Actinobacteria		0.1990	**0.047**	0.0499	0.185
	Chloroflexi		0.1593	**0.036**	0.1608	**0.038**
	Firmicutes		0.1754	**0.042**	0.0829	0.118
	Gemmatimonadetes		0.2732	**0.015**	0.2590	**0.031**
	Delta-Proteobacteria		0.2819	**0.008**	0.2746	**0.008**
	Gamma-Proteobacteria		0.3841	**0.002**	0.3045	**0.005**
	**Dothideomycetes**		0.2497	**0.009**	0.2037	**0.014**
	**Sordariomycetes**		0.3335	**0.001**	0.2825	**0.008**

## Supplementary Materials

The following are available online at https://www.mdpi.com/1660-4601/17/10/3516/s1. Table S1: Results of soil physical and chemical properties.
