# Supplementary material for: Interaction between Fungal Communities, Soil Properties, and the Survival of Invading E. coli O157:H7 in Soils
Source: Int J Environ Res Public Health. 2020 May 18;17(10):3516. doi: 10.3390/ijerph17103516 (PMC7277763; doi:10.3390/ijerph17103516)
Supplement: Supplementary file 1 [file ijerph-17-03516-s001.pdf]

Table S1. Results of soil physical and chemical properties.

sample	EC (ms/cm)	WSOC (mg/kg)	TN (mg/kg)	NH_4-N (mg/kg)	NO_3-N (mg/kg)	TP (mg/kg)	pH	clay (%)	C/N	N/P
TL1	5.66	10.00	19.31	0.44	0.84	9.89	7.85	0.07	0.52	1.95
TL2	12.45	34.43	40.69	0.56	2.39	8.58	8.49	0.70	0.85	4.74
TL3	16.07	40.50	53.26	0.79	4.92	9.23	8.44	1.54	0.76	5.77
TL4	12.29	14.18	34.74	0.25	3.15	9.41	8.35	1.52	0.41	3.69
TL5	10.68	10.13	29.48	0.42	2.08	8.41	8.57	1.59	0.34	3.50
TL6	7.36	22.28	16.09	0.39	0.89	9.27	8.33	1.15	1.38	1.74
TL7	9.38	20.25	17.51	0.48	0.80	9.30	8.55	0.78	1.16	1.88
TL8	6.64	10.13	22.34	1.23	1.11	10.23	8.01	0.00	0.45	2.18
SP1	7.41	14.18	43.66	0.22	4.17	2.74	7.58	7.85	0.32	15.91
SP2	17.24	24.30	100.38	0.81	11.80	4.81	8.02	6.70	0.24	20.87
SP3	18.23	38.48	116.38	1.18	15.48	3.37	8.03	7.10	0.33	34.56
SP4	37.70	32.40	56.52	0.30	5.81	6.43	7.87	6.33	0.57	8.79
SP5	33.00	24.30	65.31	0.40	5.90	4.17	7.31	7.04	0.37	15.66
SP6	28.90	28.35	96.44	1.36	9.45	4.14	6.78	7.74	0.29	23.27
YJ1	76.00	83.11	15.07	2.99	2.57	401.36	6.34	12.40	5.51	0.04
YJ2	195.00	91.85	24.05	4.05	9.08	1418.46	6.88	8.26	3.82	0.02
YJ3	75.00	135.59	16.08	1.32	4.48	700.50	6.88	7.60	8.43	0.02
YJ4	110.00	113.72	18.40	1.88	7.08	1106.85	6.80	8.37	6.18	0.02
YJ5	70.00	65.61	14.77	2.99	3.56	555.92	6.86	10.41	4.44	0.03
YJ6	120.00	100.60	23.90	11.91	17.71	2767.12	5.76	10.29	4.21	0.01
YJ7	80.00	91.85	17.44	7.14	7.39	1154.21	5.87	13.97	5.27	0.02

EC, electrical conductivity; WSOC, water-soluble organic carbon; TN, total soluble nitrogen; NH_4-N ammonium nitrogen; NO_3-N, nitrate nitrogen; TP, total dissolved phosphorus; clay (%), soil clay content.
